# Voltammetric Determination of 3-Methylmorphine Using Glassy Carbon Electrode Modified with rGO and Bismuth Film

**DOI:** 10.3390/bios12100860

**Published:** 2022-10-12

**Authors:** Ademar Wong, Anderson M. Santos, Camila A. Proença, Thaísa A. Baldo, Maria H. A. Feitosa, Fernando C. Moraes, Maria D. P. T. Sotomayor

**Affiliations:** 1 Institute of Chemistry, State University of São Paulo (UNESP), Araraquara 14801-970, Brazil; 2 Department of Chemistry, Federal University of São Carlos (UFSCar), São Carlos 13560-970, Brazil

**Keywords:** reduced graphene oxide, bismuth, 3-methylmorphine, electrochemical sensor, electrodeposition

## Abstract

This work reports the development and application of a simple, rapid and low-cost voltammetric method for the determination of 3-methylmorphine at nanomolar levels in clinical and environmental samples. The proposed method involves the combined application of a glassy carbon electrode modified with reduced graphene oxide, chitosan and bismuth film (Bi-rGO-CTS/GCE) via square-wave voltammetry using 0.04 mol L^−1^ Britton–Robinson buffer solution (pH 4.0). The application of the technique yielded low limit of detection of 24 × 10^−9^ mol L^−1^ and linear concentration range of 2.5 × 10^−7^ to 8.2 × 10^−6^ mol L^−1^. The Bi-rGO-CTS/GCE sensor was successfully applied for the detection of 3-methylmorphine in the presence of other compounds, including paracetamol and caffeine. The results obtained also showed that the application of the sensor for 3-methylmorphine detection did not experience any significant interference in the presence of silicon dioxide, povidone, cellulose, magnesium stearate, urea, ascorbic acid, humic acid and croscarmellose. The applicability of the Bi-rGO-CTS/GCE sensor for the detection of 3-methylmorphine was evaluated using synthetic urine, serum, and river water samples through addition and recovery tests, and the results obtained were found to be similar to those obtained for the high-performance liquid chromatography method (HPLC)—used as a reference method. The findings of this study show that the proposed voltammetric method is a simple, fast and highly efficient alternative technique for the detection of 3-methylmorphine in both biological and environmental samples.

## 1. Introduction

Over the past few decades, researchers from different fields have shown an increasingly growing interest in the determination of drugs present in clinical, environmental and pharmaceutical samples with a view to tackling the problems related to drug intoxication and wastewater contamination as well as ensuring the quality control of pharmaceutical products [1]. This has resulted in the development of several techniques with accurate tools targeted at the qualitative (simple yes/no analysis) and/or quantitative determination of drugs at trace levels in different samples (including urine, serum and water).

One of the drugs most commonly consumed for the treatment of pains is 3-methylmorphine; this opioid analgesic, which is also referred to as 3-methylmorphine (codeine), is obtained from the methylation of morphine derived from Papaver somniferum (poppy seeds) [2]. 3-methylmorphine has a sedative effect which helps to manage mild to moderate pain. Despite its proven efficiency in pain alleviation, 3-methylmorphine has some profoundly disturbing side effects; one of these side effects is that it can affect the central nervous system, causing mood swings. The analgesic effect of 3-methylmorphine can be potentiated when it is applied in combination with acetaminophen and caffeine [3].

Owing to its widespread application and rampant disposal in the environment, several studies reported in the literature have employed a wide range of analytical techniques for the determination/detection of 3-methylmorphine in different matrices; the techniques employed have included the following: colorimetry [4,5], spectrophotometry [6,7], chromatography [8,9], capillary electromigration [10,11], and electrochemical techniques [12,13]. Significant improvements have been increasingly made in the design of these techniques in order to ensure accurate quantification of drug species in complex matrices so as to avoid false positive or negative signals due to the high concentration levels of interferents in these matrices.

The use of electrochemical sensors for the detection of compounds of interest in complex matrices has attracted considerable interest among researchers because of their operational simplicity, sensibility and high degree of accuracy; in addition, the application of these sensors with rapid detection techniques such as square-wave voltammetry (SWV) allows one to identify the analytes of interest within an extremely short period of time (for instance, in seconds) [14]. The sensibility and selectivity of electrochemical sensors can be improved by modifying the devices with nanomaterials, such as reduced graphene oxide (rGO); this contributes toward enhancing the mechanical resistance of the sensors, as well as their thermal and electrical conductivity [15,16]. Furthermore, the use of suitable hybrid materials for the modification of working electrodes may help to improve the properties of the sensor. Bismuth film-based electrodes have been widely used in electroanalysis for the determination of analytes such as metals, pesticides and pharmaceutical products. Bismuth film can be used in combination with rGO in order to promote efficient synergistic effects which can effectively improve the sensor properties, leading to low residual current, good chemical stability, and high mechanical stability, apart from allowing the formation of “fused alloys” with heavy metals [17].

In the present work, we report the development and application of an electrochemical sensor, which was constructed using glassy carbon electrode (GCE) modified with rGO and bismuth film, for the determination of 3-methylmorphine. The clinical and environmental applicability of the sensor was also evaluated through the application of the device for the detection of 3-methylmorphine in synthetic urine, serum, and river water samples; the results obtained were then compared with those of HPLC used as a reference method.

## 2. Materials and Methods

### 2.1. Reagents and Apparatus

All the reagents (purity ≥ 98%) were used as purchased. 3-methylmorphine, ascorbic acid, caffeine, paracetamol, bismuth (Bi), chitosan (CTS), glutaraldehyde (Glu), KCl and bovine serum were purchased from Sigma-Aldrich (St. Louis, MO, USA). NaOH, Na_2_[B_4_O_5_(OH)_4_, (H_2_SO_4_), CH_3_COOH and H_3_BO_3_ were acquired from Synth (São Paulo, Brazil). Graphene was acquired from the Graphene Supermarket (New York, NY, USA). All solutions were prepared using ultrapure water (with resistivity ≥ 18.0 MΩ cm) acquired from a Milli-Q system (Millipore^®^). Stock solution of 0.01 mol L^−1^ 3-methylmorphine was dissolved in ultrapure water.

The electrochemical experiments were performed using Autolab PGSTAT 302N potentiostat (Herisau, Switzerland) controlled by the NOVA 2.1 software and equipped with a conventional electrochemical cell of 10.0 mL volume which consisted of the following three electrodes: Ag/AgCl (3.0 mol L^−1^ KCl) used as reference electrode (RE); platinum coil employed as counter electrode (CE); and GCE used as working electrode (WE) (r = 0.15 cm).

Morphological characterization of the materials was performed using images acquired from Supra 35-VP microscope (Oberkochen, Germany) and the confocal optical microscope (Olympus, LEXT OLS 4000) controlled via the 2.2.6 software (CA, USA).

Fourier transform infrared spectroscopy analyses of the rGO and bismuth film were performed using FTIR–Vertex 70 spectrometer (Bruker, Karlsruhe, Germany) with spectral range of 4000 to 400 cm^−1^.

The chromatographic analysis was conducted using Shimadzu model 10ATvp LC (Kyoto, Japan) system which consisted of two pumps (LC–10AT), column oven (CTO10A), and UV detector (SPD–10A). The chromatographic experiments were performed under the following conditions—mobile phase: 1% phosphoric acid (*v/v*) and acetonitrile in the ratio of 85:15 (*v/v*)); temperature: 30 °C; flow rate: 1.0 mL min^−1^; detection wavelength: 220 nm [18].

### 2.2. Preparation of rGO and Modification of GCE with rGO and Bismuth Film

Initially, graphene oxide (GO) was synthesized, as described in the literature [19], using a mixture of H_2_SO_4_/HNO_3_ concentrated in the proportion of 3:1 (*v*/*v*). Thus, an amount of 200 mg of graphene was mixed in the acidic solution, and the mixture was kept under stirring for 12 h at 25 °C. Subsequently, the GO suspension obtained was then filtered and washed with ultrapure water until a pH close to 7.0 was obtained. After that, the mixture was dried in an oven at 100 °C. A mass of 50 mg GO was diluted in ultrapure water (50 mL) to form a suspension of 1 mg/mL. Thereafter, 1.0 g NaBH_4_ and 0.5 g CaCl_2_ were added to a 50 mL GO suspension. The mixture was kept under stirring at 25 °C for 12 h to obtain the rGO. The solid material (rGO) obtained was filtered and washed with ultrapure water [20,21].

In the next step, the surface of the GCE was cleaned by polishing with alumina slurry (1.0 µm) on a polishing cloth. Subsequently, the GCE was subjected to ultrasonic bath with deionized water for 1 min. The Bi-rGO-CTS/GCE dispersion was prepared using 2.0 mg of rGO, 500 µL of CTS (0.1 % *v*/*v*), 500 µL of Glu (0.2 % *v*/*v*), and 1.0 mL of 0.01 mol L^−1^ sodium hydroxide. The material was subjected to ultrasonic agitation for 25 min. After that, 9.0 µL of the homogeneous dispersion was added on the electrode surface and was left to dry (25 °C) for 2 h. To prepare the Bi-rGO-CTS/GCE sensor, the bismuth film was electrochemically deposited on the electrode surface using an applied potential of −0.18 V vs. Ag/AgCl (3.0 mol L^−1^ KCl) for 200 s and a solution containing 0.02 mol L^−1^ Bi(NO_3_)_3_, 0.15 mol L^−1^ sodium citrate and 1.00 mol L^−1^ HCl [17].

### 2.3. Preparation of Synthetic Urine, Bovine Serum and River Water Samples

The synthetic urine sample was prepared based on the work conducted by Laube et al. [22] using 49, 10, 20, 15, 18, and 18 mmol L^−1^ of NaCl, CaCl_2_, KCl, KH_2_PO_4_, NH_4_Cl and urea, respectively. The serum samples were prepared using commercial bovine calf serum obtained from Sigma-Aldrich. River water samples were collected from the Jacaré-Guaçu river in the city of Araraquara—Sao Paulo, Brazil. The collection sites were recorded by GPS (21°51′23.3″ S 48°19′49.8″ W). The samples were subjected to conventional filtration to remove any solid material and were then stored in a 100.0 mL flask and kept in the refrigerator at 0 °C. The samples were enriched with 3-methylmorphine concentration. A dilution of 100 times was used in the electrochemical cell.

### 2.4. Analytical Procedure

First, the morphological and electrochemical characterizations of the electrodes were performed by scanning electron microscopy (SEM) and energy-dispersive X-ray (EDX).

The electrochemical oxidation of 3-methylmorphine was evaluated by cyclic voltammetry (CV) and the optimization of the experimental conditions was conducted using square-wave voltammetry (SWV). Under optimal conditions, analytical curves were constructed with the successive additions of 3-methylmorphine. The limit of detection (LOD) was calculated based on the following equation: 3 × *SD/m*, where “*SD*” stands for standard deviation for 10 blank solutions (*n =* 10) and *“m”* represents the slope. The precision and selectivity of the proposed method based on the application of the Bi-rGO-CTS/GCE sensor were evaluated through repeatability studies and analysis of potential interference. The proposed sensor (Bi-rGO-CTS/GCE) was applied for the detection of 3-methylmorphine in biological (bovine serum and urine synthetic) and environmental (river water) samples. Finally, the results obtained were compared with those of high-performance liquid chromatography (reference method).

## 3. Results and Discussion

### 3.1. Material Characterization

**Figure 1:**
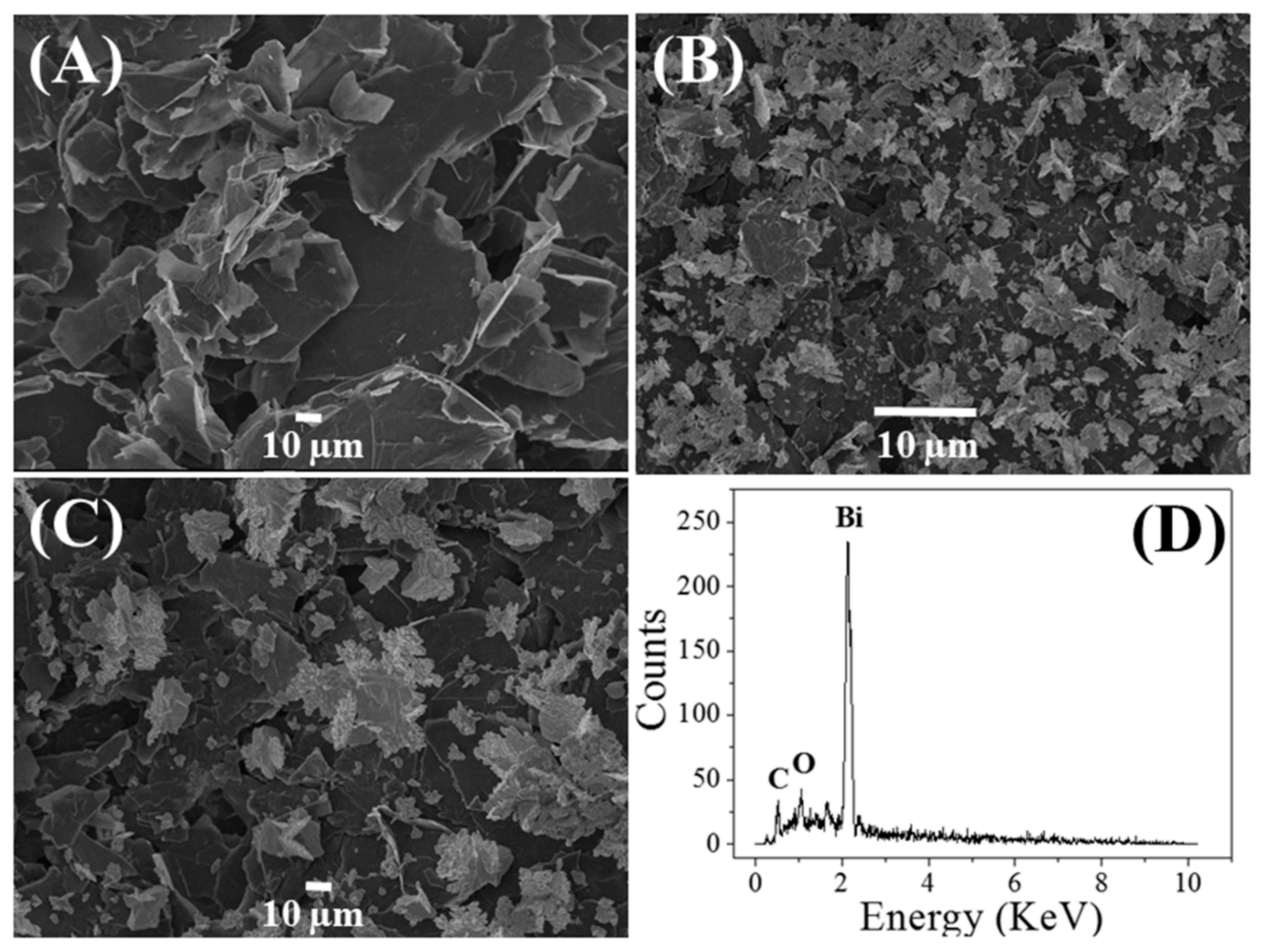
SEM images of reduced graphene oxide (**A**); bismuth on reduced graphene oxide (**B**,**C**); and EDX (**D**).

The morphology of the materials (rGO and Bi-rGO film) was characterized by scanning electron microscopy (SEM). The SEM image of the rGO material was characterized by agglomerated and thin “sheets” of different sizes (Figure 1A). Figure 1B,C show the SEM image of Bi on reduced rGO under different magnifications. As can be noted, bismuth characterized by loose leaf structure with well-defined morphology can be found uniformly distributed on both surfaces of the rGO sheets; this is clearly indicative of the successful formation of bismuth on the rGO material.

The EDX profile of the Bi-rGO material (Figure 1C) exhibited signals of carbon, oxygen and bismuth atoms; this pointed to the successful mixture of the rGO nanosheets with the Bismuth film.

**Figure 2:**
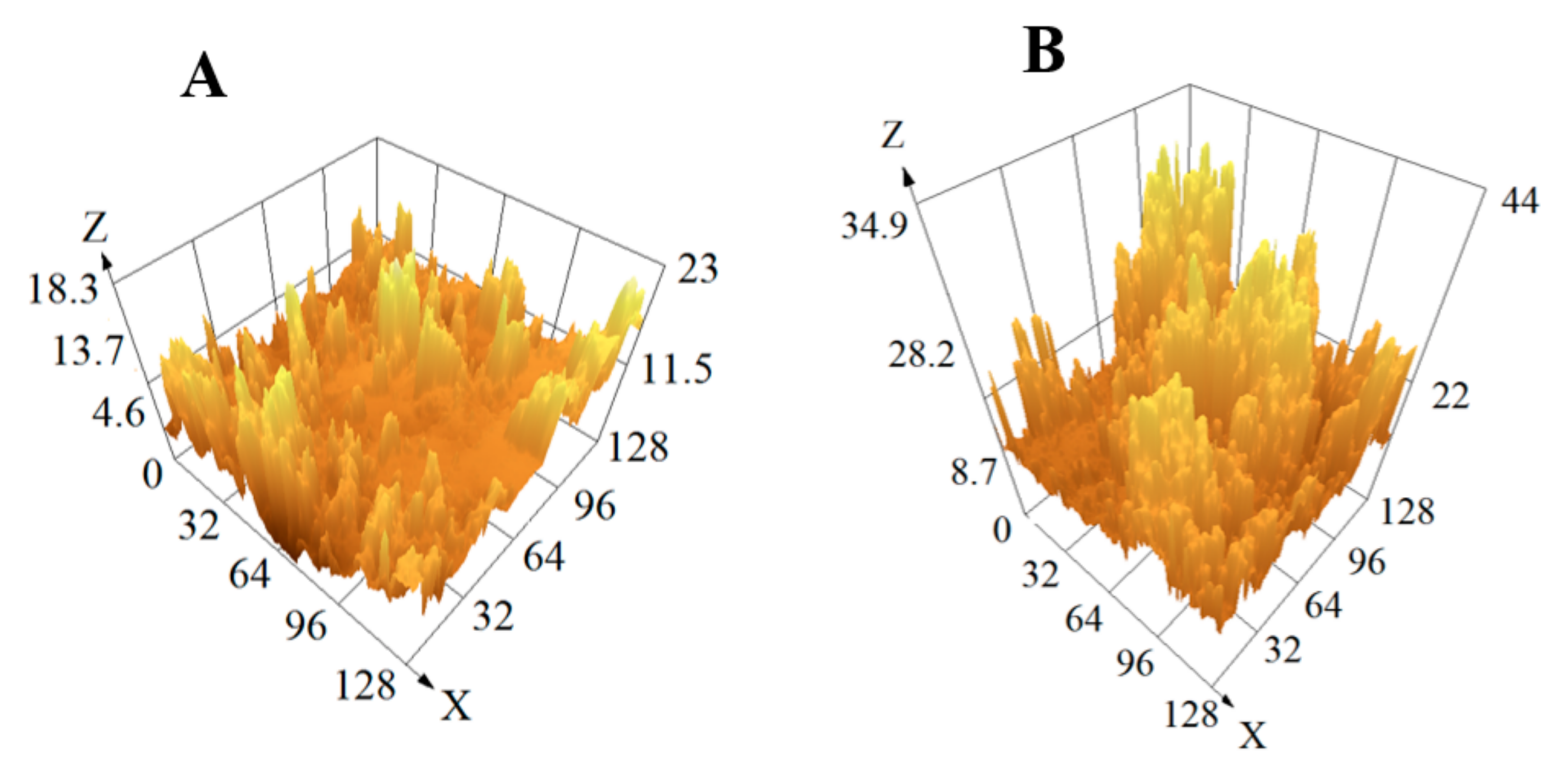
Topographic image of the (**A**) rGO-CTS and (**B**) Bi-rGO-CTS film surface obtained from confocal optical microscope (100 times of magnification). X and Z axes (scale, µm).

The confocal optical microscope (LEXT OLS 4000) was used to obtain the images of the materials in 3D imaging format with great definition. Looking at Figure 2, one can clearly see a significant difference in the topographic images of the materials incorporated onto the surface of the proposed electrode. The thickness of the films was calculated using the Olympus software. The average values obtained for the thickness of the films were as follows: 3.1 ± 0.2 μm for rGO-CTS (Figure 2A) and 5.7 ± 0.2 μm for Bi-rGO-CTS (Figure 2B) (*n* = 5).

### 3.2. Analytical Response

**Figure 3:**
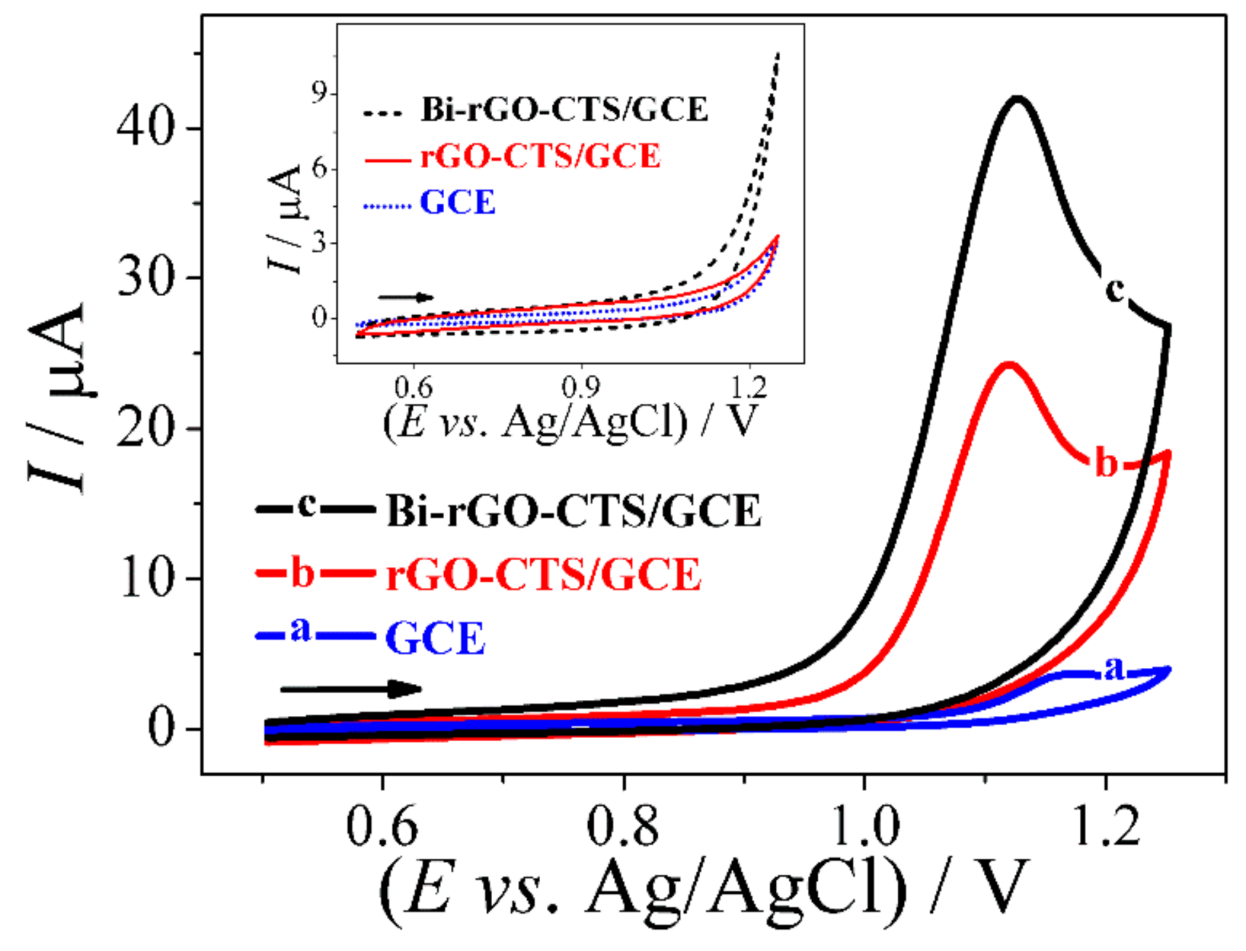
Electrochemical profiles of GCE, rGO-CTS/GCE and Bi-rGO-CTS/GCE. Analysis conditions-electrolyte solution: 0.04 mol L^−1^ Britton-Robinson buffer solution (pH 4); scan rate (ν): 50 mV s^−1^; 3-methylmorphine concentration: 0.10 mmol L^−1^. Inset: without the addition of 3-methylmorphine.

Cyclic voltammetry plots were used to monitor the electrochemical profile of the modified electrodes. Figure 3 shows the electrochemical response (potential range: 0.5 V to 1.25 V, and ν = 50 mV s^−1^) of the following electrodes: GCE, rGO-CTS/GCE and Bi-rGO-CTS/GCE in the presence of 3-methylmorphine and without the addition of 3-methylmorphine (inset).

Figure 3a shows that the bare GCE did not exhibit any significant electrochemical response (voltammetric response) in the presence of 0.10 mmol L^−1^ standard solution of 3-methylmorphine. Figure 3b,c show the electrochemical profiles obtained for rGO-CTS/GCE and Bi-rGO-CTS/GCE, respectively, in the presence of a solution containing 3-methylmorphine; as can be observed, both electrodes exhibited high electrochemical response with well-defined irreversible oxidation peaks at +1.1 V, corresponding to the oxidation of 3-methylmorphine on the surface of the electrodes. The current values recorded for the bare GCE, rGO-CTS/GCE and Bi-rGO-CTS/GCE were 2.6 µA, 22 µA, and 39 µA, respectively. This result clearly points to the significant role played by Bi and rGO in the direct electrochemical detection of 3-methylmorphine. The analysis of the parameters mentioned above helped confirm that the modification of the electrode with Bi film led to an increase in both the surface area and the faradaic current.

**Figure 4:**
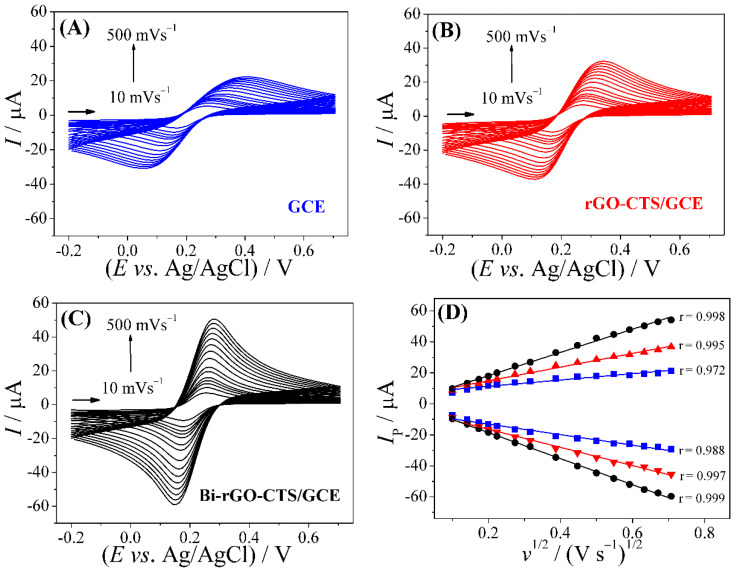
Analysis of the scan rate of the (**A**) GCE, (**B**) rGO-CCTS/GCE and (**C**) Bi-rGO-CTS/GCE electrodes using 0.1 mol L^−1^ KCl solution in the presence of redox probe [Fe(CN)_6_]^3−/4^^−^ (1.0 mmol L^–1^) at different potential scan rates (10–500 mV s^−1^). (**D**) *I*_p_ vs. ν^1/2^.

Electrochemical studies were carried out in order to evaluate the electrochemical properties of the electrodes prepared in this study; with the aid of the electrochemical probe [Fe(CN)_6_]^3−/4−^ and through the application of cyclic voltammetry (CV), we were able to analyze the reversibility of the redox behavior, the change in conductivity, and the active surface area of the electrodes-see Figure 4. The scan rate of the bare GCE (Figure 4A), rGO-CTS/GCE (Figure 4B) and Bi-rGO-CTS/GCE (Figure 4C) was evaluated using 0.1 mol L^−1^ KCl as electrolyte solution with the redox probe [Fe(CN)_6_]^3−/4−^ (1.0 mmol L^–1^) at scan rate of 10 to 500 mV s^−1^.

The Randles–Sevcik equation (Equation (1)) was used to calculate the active surface area of the sensors. The CVs obtained exhibited linear relationships between the anodic peak current (*I*_p_) and the square root of the scan rate (ν^1/2^).

*I*_p_ = (2.69 × 10^5^) *ACD*^1/2^*n*^3/2^ν^1/2^ (1)

In Equation (1), *I*_p_ is the peak current (A), *A* is the electroactive area (cm^2^), *n* is the number of electrons transferred, *D* is the diffusion coefficient of [Fe(CN)_6_]^3–/4-^ in the 0.10 mol L^−1^ KCl solution (7.6 × 10^−6^ cm^2^ s^−1^), ν is the potential scan rate (V s^−1^), and *C* is the [Fe(CN)_6_]^3−^ concentration (mol cm^−3^).

The surface area of the electrodes was calculated from the slope of *I*_p_ vs. ν^1/2^ (Figure 4D). The active surface area obtained for the bare GCE, rGO-CTS/GCE and Bi-rGO-CTS/GCE sensor was 0.041 cm^2^, 0.077 cm^2^ and 0.119 cm^2^, respectively.

The heterogeneous electron transfer rate constant (*k*^0^) was used to evaluate how the charge transfer kinetics is affected by the sensor modification. The rate constant values were found using the Nicholson Equation (2) [23].

Ψ = *k*^0^ [π*Dn*ν*F*/(*RT*)]^−1/2^ (2)

In Equation (2), Ψ is the kinetic parameter, π = 3.1415, *T* = 298 K, and *F* and *R* are the Faraday (96,485 C mol^−1^) and universal gas (8.314 J K^−1^ mol^−1^) constants. The Ψ values were obtained as proposed by Lavagnini et al. [24], according to Equation (3), which correlates Ψ with Δ*E*_p_.

Ψ = (−0.6288 + 0.0021 *n*Δ*E*_p_)/(1 − 0.017 *n*Δ*E*_p_) (3)

With regard to the above equation, the slope of the equation of the line directly represents the value (obtained) of *k*^0^, which in this case was equal to 1.7 × 10^−3^ cm s^−1^ for GCE, 3.8 × 10^−3^ cm s^−1^ for rGO-CTS/GCE, and 8.9 × 10^−3^ cm s^−1^ for Bi-rGO-CTS/GCE. Thus, the *k*^0^ value obtained for Bi-rGO-CTS/GCE was about 5.2 times greater than that obtained for GCE. This result points to an improvement in the electronic transfer speed for Bi-rGO-CTS/GCE compared to the other electrodes.

**Figure 5:**
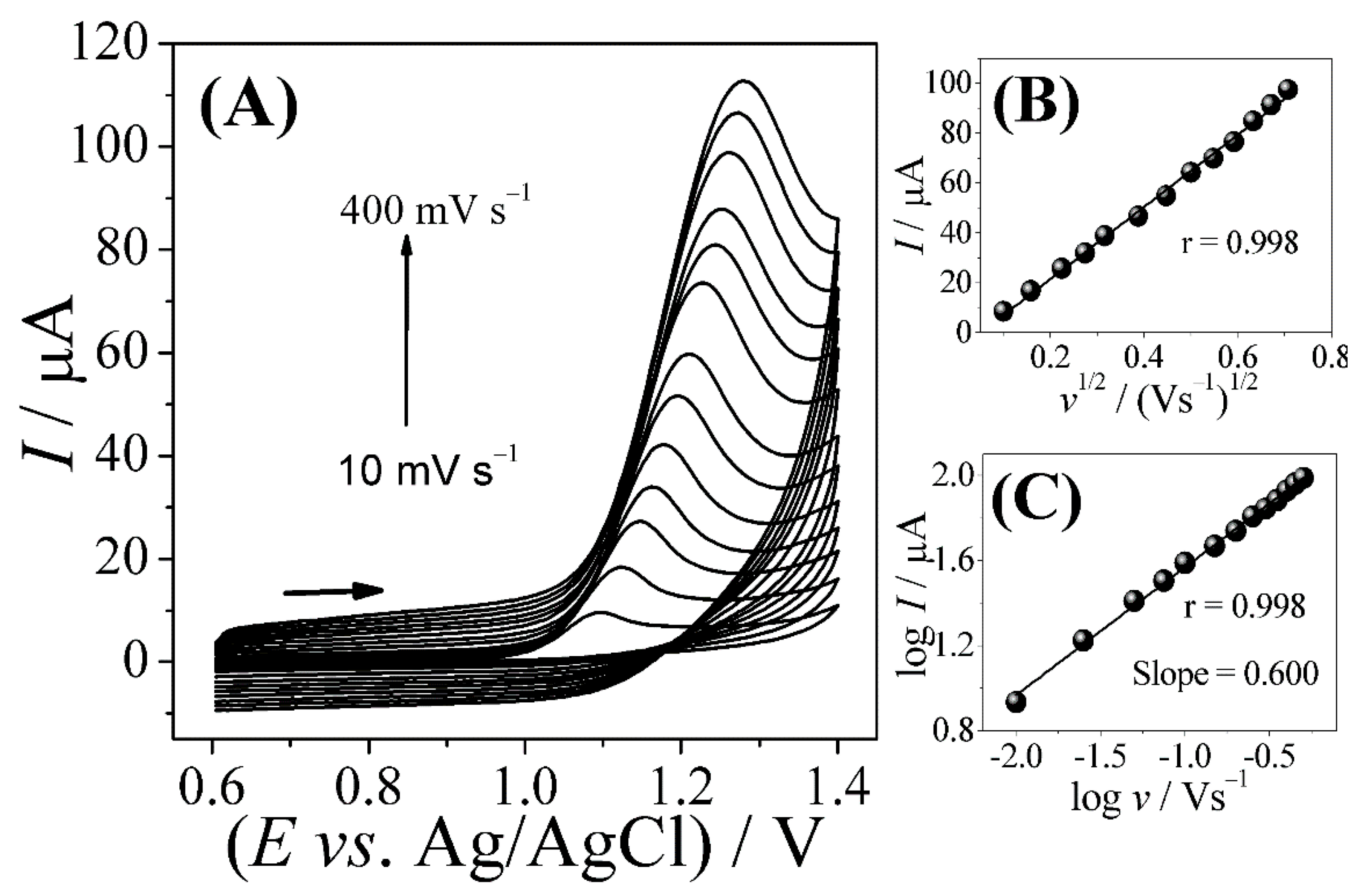
(**A**) Cyclic voltammograms at different scan rates (10–400 mV s^−1^) based on the application of the Bi-rGO-CTS/GCE sensor in 0.20 mol L^−1^ phosphate-buffered solution (pH 6.0) in the presence of 50.0 µmol L^−1^ concentration of 3-methylmorphine. Plots: (**B**) *I*_pa_ vs. ν^1/2^ and (**C**) log *I*_pa_ vs. logν.

A thorough analysis was also conducted in order to evaluate the influence of scan rate on the analytical response using 50.0 µmol L^−1^ 3-methylmorphine in phosphate-buffered solution (pH 6.0) and scan rates ranging from 10 to 400 mV s^−1^ (Figure 5A). The anodic peak currents (*I*_pa_) of 3-methylmorphine in the phosphate-buffered solution (pH 6.0) shifted to more positive values as the scan rate increased; this is clearly indicative of an irreversible electrochemical reaction. The *I*_pa_ vs. ν^1/2^ plot was found to be linear (Figure 5B); this shows that the mass transport of the 3-methylmorphine on the electrode surface occurred by diffusion. Plots of log *I*_pa_ vs. log ν (Figure 5C) exhibited a slope of 0.60, which is typically characteristic of systems controlled by diffusion.

Using the cyclic voltammograms obtained at different potential scan rates, we were able to calculate the average value of *E*_p_ − *E*_1/2_ (see Equation (4)). By inserting the value obtained (64 mV), we obtained the relation αn = 0.75. Considering α = 0.50 (value normally attributed to irreversible processes), we estimated the number of electrons (*n*) involved in the 3-methylmorphine irreversible oxidation on the electrode surface. The value of (*n*) was found to be approximately 1.5 (close to 2); this value is similar to the values obtained in other studies reported in the literature [25].

(4) $$│E_{p}-E_{1/2}│=\frac{48\;\;}{\alpha n}\;$$

In Equation (4), *E*_p_ stands for peak potential, and *E*_1/2_ is the half-wave potential obtained from the cyclic voltammetry graph (Figure 5).

**Figure 6:**
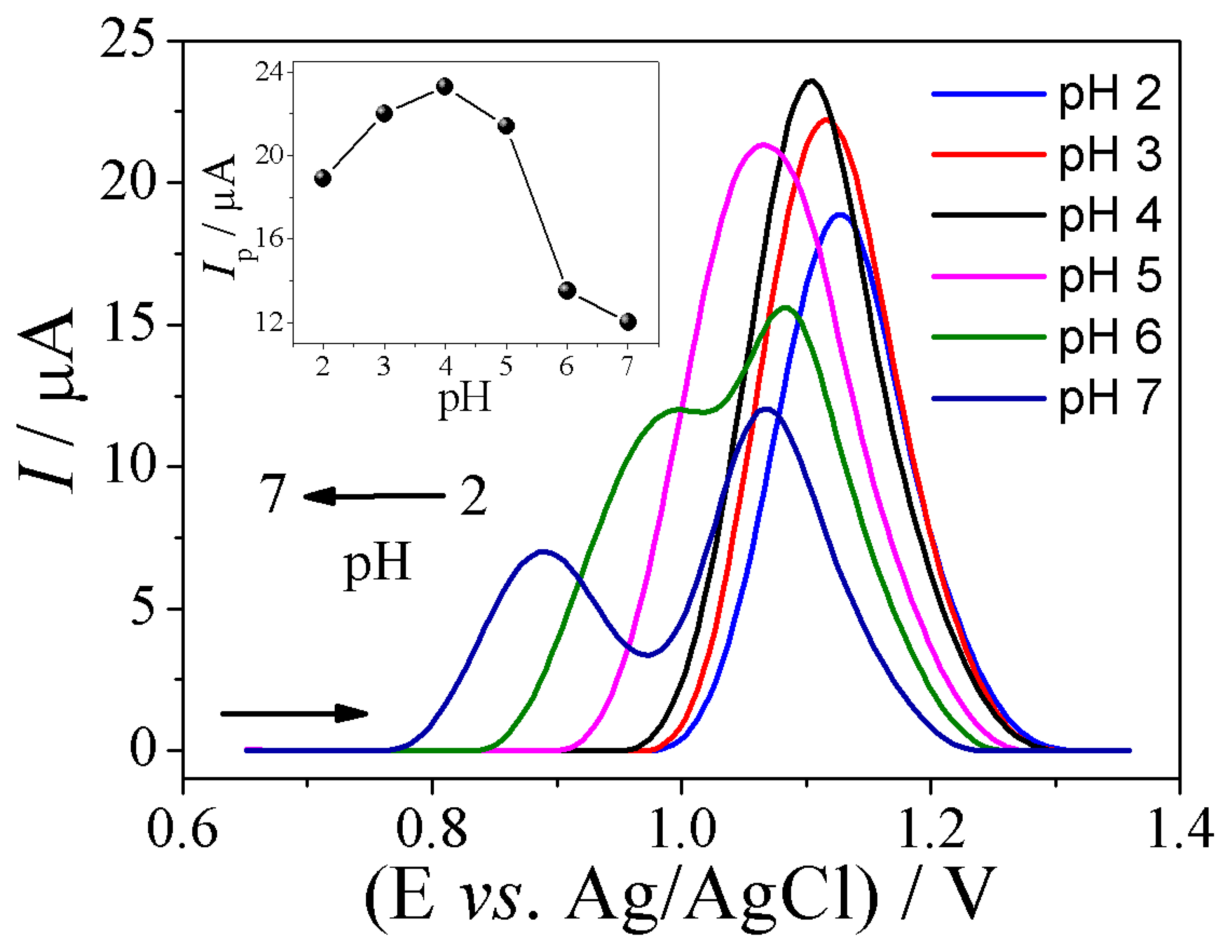
Results obtained from SWV analysis conducted based on the application of the Bi-rGO-CTS/GCE sensor in 0.04 mol L^−1^ Britton–Robinson buffer solution containing 5.0 µmol L^−1^ of 3-methylmorphine at pH ranging from 2.0 to 7.0. Parameters: *f* = 15 Hz; *a* = 50 mV; Δ*E*_s_ = 5 mV. Inset: *I*_p_ vs. pH plots.

The analysis of the influence of pH on the peak potential and the peak current related to 3-methylmorphine oxidation was conducted by square-wave voltammetry using 0.04 mol L^−1^ Britton–Robinson buffer solution (pH range of 2.0–7.0) as supporting electrolyte. As can be observed in Figure 6, a decrease in hydrogen ionic concentration of the electrolyte was found to cause a shift in peak potential for 3-methylmorphine oxidation toward less positive values; this outcome is attributed to deprotonation during the oxidation process, which is facilitated at higher pH values.

**Scheme 1:**
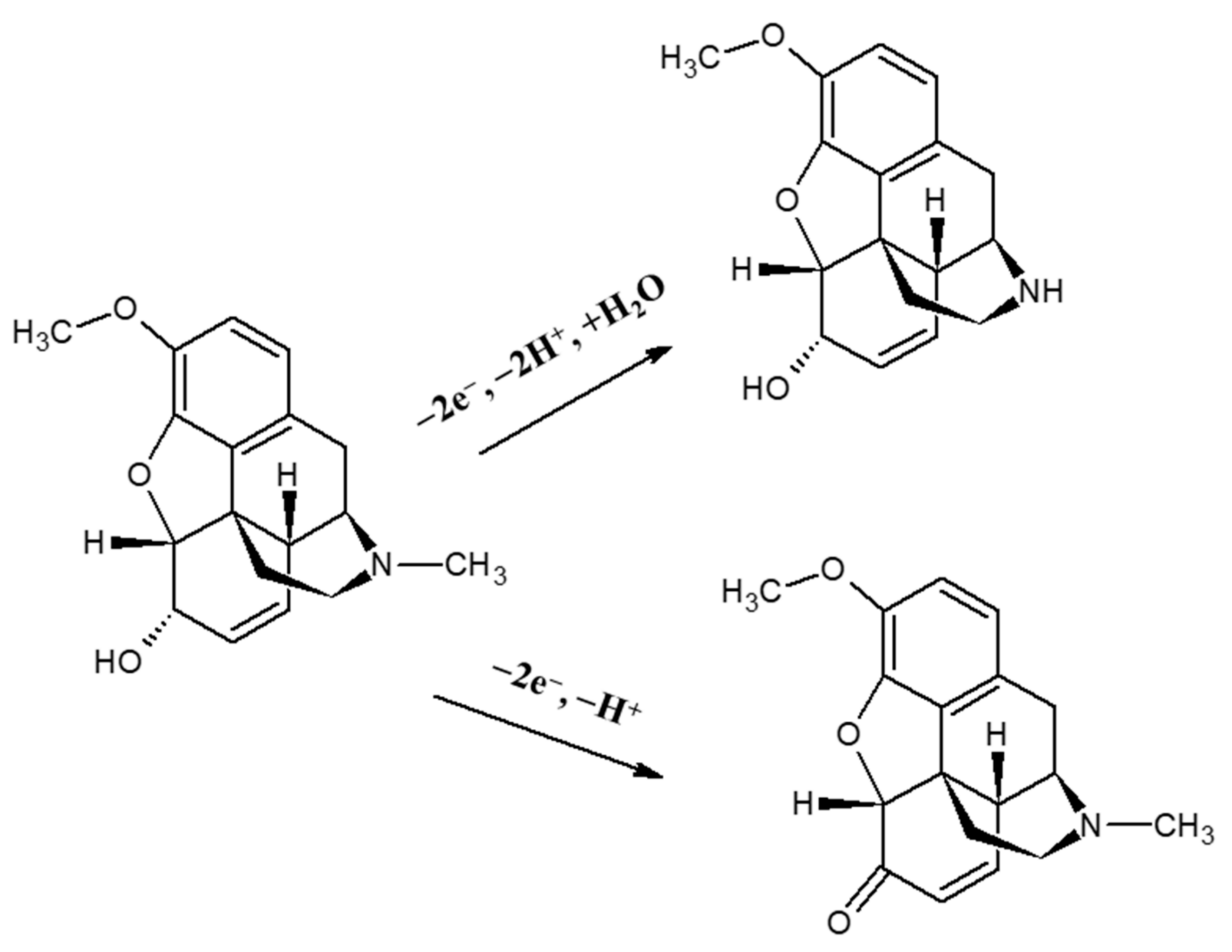
Proposed mechanism for the electro-oxidation of 3-methylmorphine [26].

Additionally, as can be verified, a single anodic peak was observed in the pH range of 2.0 to 5.0, and two anodic peaks were detected at pH = 6.0 and 7.0. Where peak one can be associated with the oxidation of the 6-hydroxy group with loss of 2 electrons and 1 proton, and peak two (varying of 0.95 V and 0.88 V) can be associated with the oxidation of the tertiary amine group involving the loss of 2 electrons and 2 protons [26]. For illustration purposes, Scheme 1 shows the electrochemical processes, which correspond to the oxidation processes of 3-methylmorphine [26].

The *I*_pa_ vs. pH plots (Figure 6 inset) for 3-methylmorphine shows an increase in the anodic peak current in the pH range of 2.0 to 4.0, with maximum peak current recorded at pH 4.0. The maximum value of the oxidation peak current for 3-methylmorphine was observed at pH 4.0; the value was found to decrease after that. In addition, an increase in pH from 2.0 to 7.0 led to a gradual shift in the oxidation peak potential to negative potential; this evidently showed that the electrochemical process was dependent on pH. Based on a comparison of the peak currents of 3-methylmorphine at different pH values, pH 4.0 was selected as the optimal pH level for the conduct of further experiments.

### 3.3. Calibration Curve

**Figure 7:**
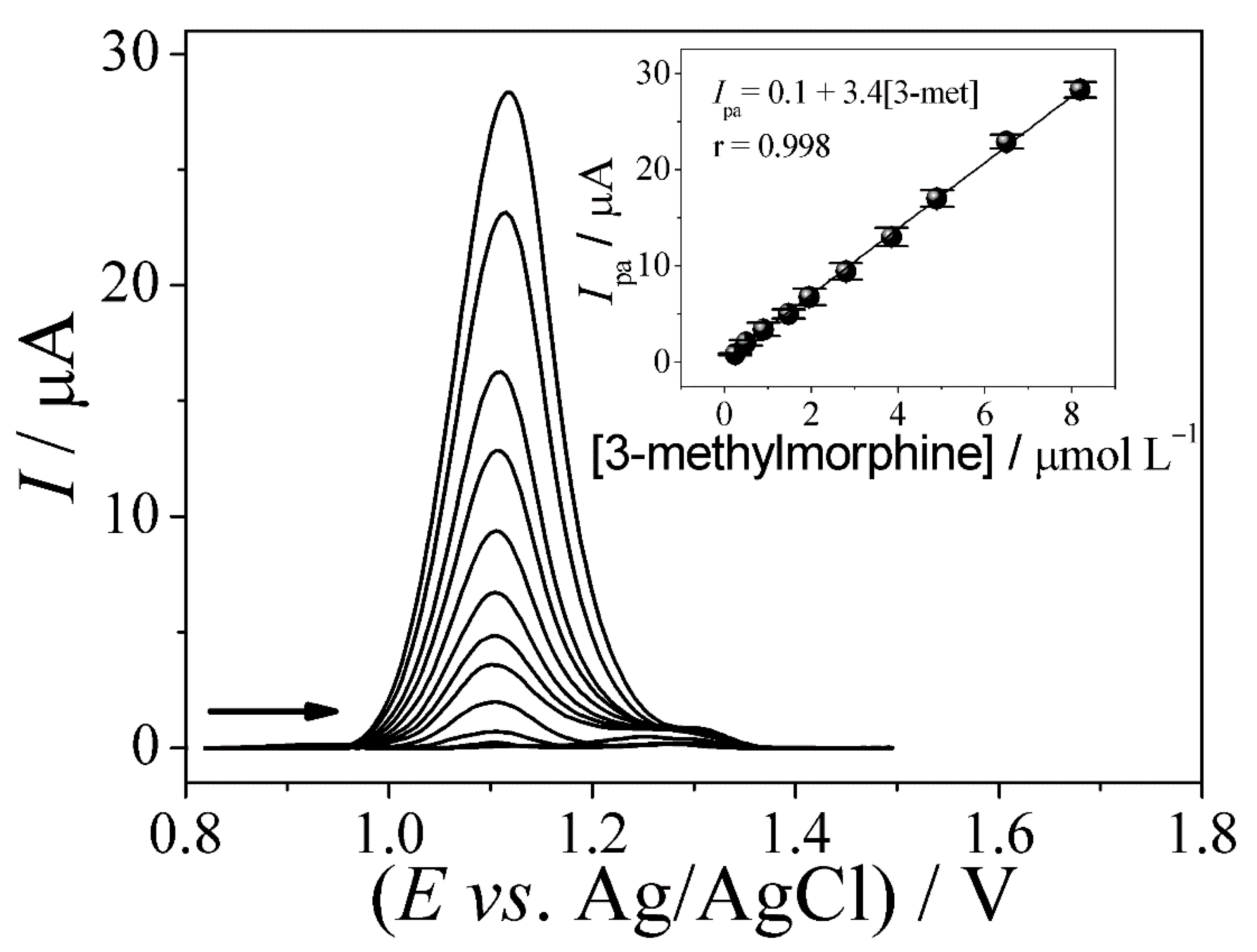
SW voltammograms obtained from the application of Bi-rGO-CTS/GCE for the determination of 3-methylmorphine (concentrations ranging from 2.5 × 10^−7^ to 8.2 × 10^−6^ mol L^−1^) in 0.04 mol L^−1^ Britton–Robinson buffer solution (pH 4.0). Inset: Corresponding calibration plots related to 3-methylmorphine concentrations. Parameters: *f* = 15 Hz; *a* = 50 mV; Δ*E*_s_ = 5 mV.

Under optimized experimental conditions, an increase in 3-methylmorphine concentration was found to lead to an increase in peak current (Figure 7). Figure 7 shows the SW voltammograms obtained from the application of Bi-rGO-CTS/GCE for the determination of 3-methylmorphine at concentrations ranging from 2.5 × 10^−7^ to 8.2 × 10^−6^ mol L^−1^ in 0.04 mol L^−1^ Britton–Robinson buffer solution (pH 4.0). The inset in Figure 7 shows the calibration plots that correspond to 3-methylmorphine concentrations. As can be observed, the calibration curve between peak current and 3-methylmorphine concentration exhibits a linear relationship (in the linear range) based on the following equation: *I*_p_ (µA) = 0.1 + 3.4 [3-methylmorphine], with correlation coefficient of 0.998. The LOD value obtained from the application of the proposed Bi-rGO-CTS/GCE was 24 × 10^−9^ mol L^−1^ (in triplicate experiments). Table 1 presents a comparative analysis of the LOD value obtained in this study and the values obtained in other studies reported in the literature related to the application of electrochemical sensors. As can be noted, the LOD value obtained in this study was satisfactorily comparable and, in some cases, better than the values reported in the literature; essentially, this points to the efficiency and viability of the method proposed in this study.

**Figure 8:**
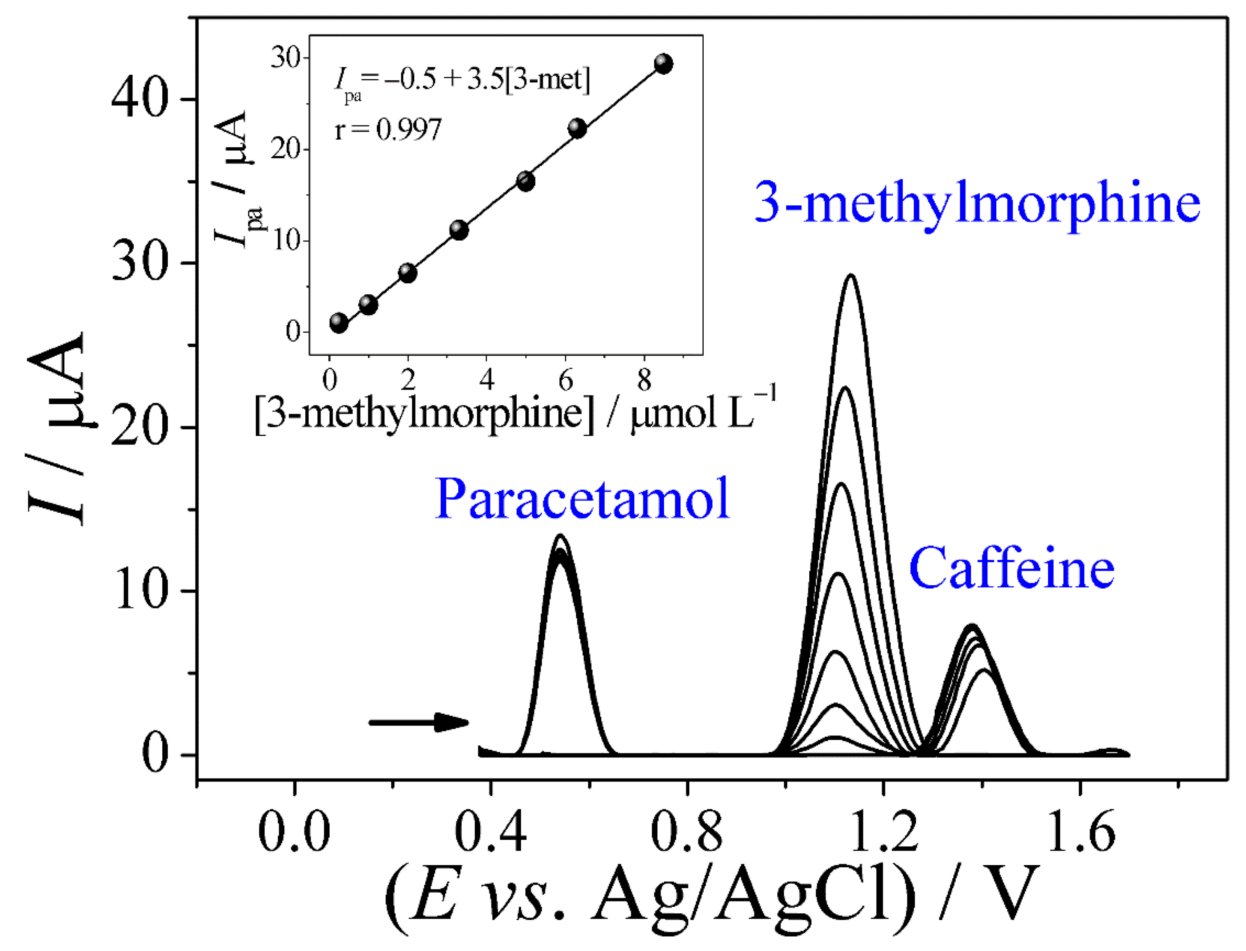
SW voltammograms obtained from the application of Bi-rGO-CTS/GCE for the determination of 3-methylmorphine in the presence of paracetamol and caffeine (at fixed concentration). Inset: Corresponding calibration plots related to 3-methylmorphine concentrations.

To evaluate the selectivity of the Bi-rGO-CTS/GCE sensor in terms of 3-methylmorphine determination, a thorough analysis was performed in order to analyze the influence of some common interfering species. Under optimized conditions, the SWV experiments were performed in the potential range of 0.35 to +1.7 V vs. Ag/AgCl (3.0 mol L^−1^ KCl) using 0.1 mol L^−1^ phosphate buffer (pH 6.0), with sequential addition of 3-methylmorphine concentration in the range of 2.5 × 10^−7^ to 8.5 × 10^−6^ mol L^−1^. The results obtained from the SWV experiments are shown in Figure 8. In all the SWV experiments, no overlap was observed between the 3-methylmorphine oxidation peak and the oxidation peaks of the interfering species. Based on the application of the proposed Bi-rGO-CTS/GCE sensor, the oxidation peak potentials obtained for paracetamol, 3-methylmorphine and caffeine were +0.50, +1.1, and +1.4 V, respectively.

Based on the comparative analysis, the linear concentration range and LOD obtained from the application of the Bi-rGO-CTS/GCE-based method was found to be comparable and sometimes better than those obtained in other studies reported in the literature.

A wide range of studies reported in the literature have employed similar oxidation reaction processes for the direct detection of 3-methylmorphine [27,28,29,30,31,32]. Carbon electrodes such as carbon paste, glassy carbon, and boron-doped diamond (BDD) have been mostly employed for the detection analysis (Table 1). Compared to metallic electrodes, the wider potential window exhibited by carbon electrodes allows one to effectively monitor redox species like 3-methylmorphine. This wider potential window is also desirable for the simultaneous determination of multiple analytes.

### 3.4. Study of Repeatability and the Influence of Possible Interferents

The analysis of repeatability of the Bi-rGO-CTS/GCE was conducted using 3.0 × 10^−6^ mol L^−1^ concentrations of 3-methylmorphine in 0.04 mol L^−1^ Britton–Robinson buffer solution (pH 4.0). As shown in Figure S1 (Supplementary Material), the RSD value obtained in 20 replicates was 3.1 % for 3-methylmorphine. The high repeatability of the proposed Bi-rGO-CTS/GCE can be attributed to the homogeneity of the electrode surface and the good conductivity derived from the application of Bi-rGO-CTS/GCE.

In addition, the influence of possible interferents, including silicon dioxide, povidone, cellulose, starch, croscarmellose, magnesium stearate, urea, ascorbic acid, and humic acid, in the ratio of 1:1 (analyte:possible interferent) was investigated. Based on the voltammograms obtained, the interferents were found to exert no influence on the determination of the analytes investigated.

### 3.5. Analysis of 3-Methylmorphine in Synthetic Urine, Bovine Serum and River Water Samples

The Bi-rGO-CTS/GCE sensor was applied for the quantification of 3-methylmorphine in samples of synthetic urine, bovine serum (commercial serum), and river water collected from the Jacaré-Guaçu River, Araraquara—São Paulo, Brazil; the samples were prepared as described in the experimental section. 3-methylmorphine determinations were performed in triplicate (*n* = 3) without any pre-treatment procedure. As can be observed in Table 2, the application of the proposed SWV-based method for 3-methylmorphine determination led to recovery percentages ranging from 95 % to 105 %.

Finally, the results obtained from the application of the two methods were compared using the paired Student’s *t* test (95 % confidence level). For this study, the calculated experimental values (2.6) were found to be lower than the *t*_critical_ value (0.2); that is, there was no significant difference between the results obtained from the two analytical methods. Thus, based on these results, it can be concluded that the proposed Bi-rGO-CTS/GCE-based method had no matrix effect; this clearly points to the great potential of the proposed technique when applied for the determination of 3-methylmorphine in urine, bovine serum and river waste samples.

## 4. Conclusions

The present work reported the successful development and application of Bi-rGO-CTS/GCE sensor for 3-methylmorphine detection. The modification of the working electrode (GCE) with rGO and bismuth film led to significant improvements in the electrochemical properties. The proposed sensor was successfully employed for 3-methylmorphine detection in synthetic urine and serum samples as well as in river water, and excellent recovery percentages were obtained (indicating the accuracy of the method). The results obtained in this study also showed that the Bi-rGO-CTS/GCE sensor has good repeatability, reproducibility and selectivity, and has great potential for application as an efficient tool for the analytical determination of 3-methylmorphine and other drugs in both biological and environmental samples.

## Figures and Tables

**Table 1:** Comparison of analytical parameters in the determination of 3-methylmorphine on different modified electrodes.

Electrode	Method	Linear Range (µmol L^−1^)	LOD (µmol L^−^^1^)	Sensitivity (µA mol^−^^1^ L)	Presence	Ref.
CD-HPC/CBPE ^1^	SWV	0.5–38	0.0095	0.047	Dipyrone	[13]
AChE-TTF-SPCEs ^2^	Chrono-amperometry	20–200	0.02	No	-	[27]
Zn_2_SnO_4_—GO/CPE ^3^	DPV	0.02–15	0.009	2.938	Morphine	[28]
ND-DHP/GCE ^4^	SWV	0.299–10.8	0.0545	0.0745	-	[29]
BDD ^5^	DPV	0.1–60	0.08	0.155	-	[30]
HTP-MWCNT/CPE ^6^	DPV	0.2–34.1 34.1–844.7	0.063	0.0288 0.009	Acetaminophen	[31]
CYP2D6-SPCEs ^7^	Chrono-amperometry	5.0–35.0	4.9	-	-	[32]
Bi-rGO-CTS/GCE	SWV	0.25–8.2	0.02	3.5	Paracetamol and caffeine	**This work**

^1^ Carbon black paste electrode modified with α-cyclodextrin and hierarchical porous carbon. ^2^ Tetrathiafulvalene/AChE- modified screen-printed carbon. ^3^ Carbon paste electrode (CPE) modified with Zn_2_SnO_4_-graphene nanocomposite. ^4^ Nanodiamond/dihexadecyl phosphate-modified glassy carbon. ^5^ Boron-doped diamond film electrode. ^6^ 4-hydroxy-2-(triphenylphosphonio)phenolate (HTP) and multiwall carbon nanotubes paste electrode. ^7^ CYP2D6-modified screen-printed carbon.

**Table 2:** Results obtained from the analysis of river water, serum and artificial urine samples.

Samples	[3-Methylmorphine]/mol L^−^^1^			Recovery ** (Sensor, %)
	Added	Found *	Comparative Method *	
Synthetic urine	1.0 × 10^−^^7^	(1.02 ± 0.08) × 10^−^^7^	(1.05 ± 0.05) × 10^−^^7^	102
	1.0 × 10^−^^6^	(0.99 ± 0.06) × 10^−^^6^	(0.95 ± 0.04) × 10^−^^7^	99.0
Serum	1.0 × 10^−^^7^	(1.03 ± 0.09) × 10^−^^7^	(1.02 ± 0.04) × 10^−^^6^	103
	1.0 × 10^−^^6^	(1.05 ± 0.07) × 10^−^^6^	(1.03 ± 0.06) × 10^−^^6^	105
River water	1.0 × 10^−^^7^	(1.00 ± 0.09) × 10^−^^7^	(1.00 ± 0.03) × 10^−^^7^	100
	1.0 × 10^−^^6^	(0.95 ± 0.06) × 10^−^^6^	(0.98 ± 0.05) × 10^−^^6^	95.0

* Average of 3 measured concentrations; ** Recovery percentage = [Found]/[Added] × 100.

## Data Availability

Not applicable.

## Supplementary Materials

The following supporting information can be downloaded at: https://www.mdpi.com/article/10.3390/bios12100860/s1, Figure S1: Repeatability study of 3-methylmorphine with the Bi-Rgo-CTS/GCE sensor in 0.04 mol L^−1^ Britton-Robinson (pH 4.0). SWV parameters: *f* = 15 Hz, *a* = 50 mV, Δ*E*s = 5 mV.
