# Supplementary material for: Voltammetric Determination of 3-Methylmorphine Using Glassy Carbon Electrode Modified with rGO and Bismuth Film
Source: Biosensors (Basel). 2022 Oct 12;12(10):860. doi: 10.3390/bios12100860 (PMC9599292; doi:10.3390/bios12100860)
Supplement: Supplementary file 1 [file biosensors-12-00860-s001.zip › biosensors-1935512-supplementary.pdf]

SUPPLEMENTARY MATERIAL

Voltammetric Determination of 3-Methylmorphine Using Glassy Carbon Electrode Modified with rGO and Bismuth Film

Ademar Wong [1], Anderson M. Santos [2], Camila A. Proença [2], Thaísa A. Baldo [2], Maria H. A. Feitosa [2], Fernando C. Moraes [2,*] and Maria D. P. T. Sotomayor [1]

[1] Institute of Chemistry, State University of São Paulo (UNESP), 14801-970 Araraquara, Brazil
[2] Department of Chemistry, Federal University of São Carlos (UFSCar), 13560-970 São Carlos, Brazil

* Correspondence: fcmoraes@ufscar.br

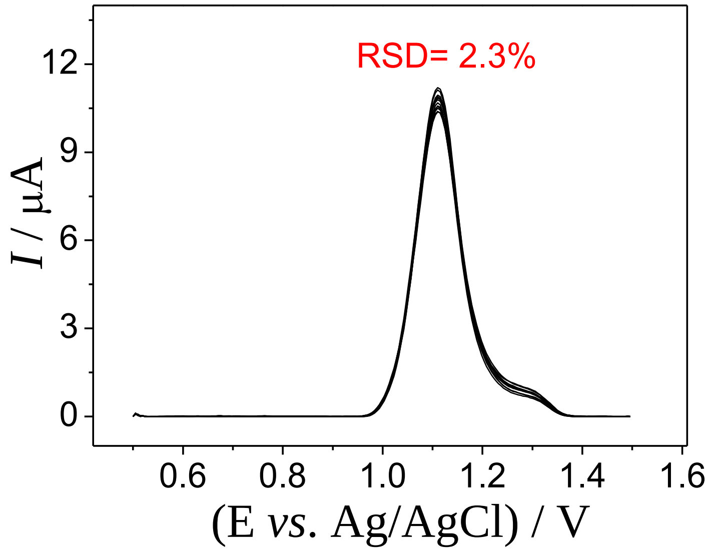

Figure S1. Repeatability study of 3-methylmorphine with the Bi-rGO-CTS/GCE sensor in 0.04 mol L^{-1} Britton-Robinson (pH 4.0). SWV parameters: f = 15 Hz, a = 50 mV, ΔE_s = 5 mV.
